# Crude Science

**Published:** 1890-12-06

**Authors:** 


### The Hospital

A Weekly Journal of

**Science, Medicine, Nursing, and Philanthropy.**

*For Hospitals, Asylums, Infirmaries, Dispensaries, Medical Practitioners, Students, Nurses, and the Charitable Public.*

Vol. IX.—No. 219.] [December 6, 1890.

### CRUDE SCIENCE.

Drs. Cornil and Babes have just issued the third edition of their work on Bacteriology. The *British Medical Journal*, in a favourable review of the book, finds it desirable to speak thus: "We regret that the authors did not think it necessary to more carefully criticise the various papers which they so frequently quote. In many instances it is impossible to know what the student is to believe, as the authors quote contradictory statements made by various authors without attempting to reconcile them." A certain class of medical writers employ the crudest style of all men that aspire to the dignity of print. Literary physicians have been known who have professed to set small store by the reasoned conclusions of metaphysicians. But the average metaphysician has at least these merits: he strictly defines his terms; he makes an effort to use them always in the same sense; and he expresses his ideas with grammatical accuracy and logical precision. These are things which a certain style of medical writer cannot, or at least does not, do. The man who is compelled to read many medical books ought to have the patience of a philosopher and the sweetness of a saint. There is generally at least one meaning on a page of any given medical book: but the reader often finds it quite impossible to discover either what it is or where it is. Not seldom he has a shrewd suspicion that the writer himself did not know either what or where his meaning was when he penned that particular page.

But medicine is a practical art: and as a science deals with things, not words; with concrete substances, and not with abstract generalisations. If it is possible to have clearness of mind at all, it is possible to have it about things. A thing is a definite object, and definite statements may be made about it. It is, for example, large or small, heavy or light, coloured or colourless, alive or dead. There is not the slightest excuse for any man to be in any confusion of mind about it: that is to say, if he has any real or positive knowledge of it. When, therefore, we find in a medical writer not only some confusion of thought, but hardly anything else but confusion, we are compelled to believe that he does not know well what he is talking about, and is only trying to persuade himself and other people that he knows.

There are those who will ask, What is the use of an abstract discussion of the demerits of medical literature? We are not writing an abstract discussion: we are making a practical protest. There are a great many medical practitioners and a large number of medical students who are always in a condition of eager quest after knowledge. They are painfully conscious of the elementary and ineffective character of much of the practice of the times. They want to get at undoubted facts, sound principles, and a rational and effective practice. They feel that there are undoubted facts to be got at, that there are sound principles, and that a rational and effective practice is possible. But the man of the bedside has neither the time nor the means of exploring with thoroughness the regions of pathology and minute anatomy, and the physiological effects of drugs. He does his part of the great work of medicine in his own place, and the scientific explorers and experimenters must do theirs. They claim great honours for themselves, and high distinctions. On what ground are they entitled to those honours and distinctions if they can neither themselves clearly understand what they are doing, nor make anybody else understand it?

The particular book, to the authors of which the *British Medical Journal* has offered such a mild and gentle remonstrance, is an example of nearly all the books which are written on the deeper subjects which enter into modern medicine. This book is designed for medical men and students, and its purpose is entirely practical. It is impossible that the authors should have designed it, like a novel or a play, for the amusement of a leisure hour. And yet the effect upon the reviewer on reading the book is to make him "confound" it in his heart as a thing impossible to be understood. He says that people who read it will not know what to believe. In saying that he is at least partially mistaken. For the reader who has any experience at all of general literature will very decidedly believe this: that the authors are very stupid persons from the literary point of view, whatever they may be from the scientific. And he will will not be slow to infer that where there is so much confusion, obscurity, and doubt in the logic, there is not likely to be much clearness and certainty in the facts with which the logic deals.

One of the worst and most exasperating vices of all medical literature is the unpardonable habit of multiplying quotations which medical authors think it right to indulge in. A very slight acquaintance with such authors is sufficient to establish this conviction in the mind, that not one in a hundred of them has any definite beliefs of his own. He seldom brings forward a single proposition, even of the most elementary kind, without buttressing it by a dozen or a score quotations from other medical writers in every part of the world. It is as if a bricklayer, when he puts a brick into its place in the wall, should apply twenty other bricks to it at different angles to prevent it falling out. A fine sample of the skilful handicraftsman he would be! Some people, it is true, have hinted that doctors do not fill their books with quotations from other doctors for the purpose of buttressing their own peculiar opinions, but with the object of showing the profound nature and wide extent of their learning. Can this be possible? Is it the case that a whole body of cultured and capable men can give themselves up to such a feeble, futile, and contemptible vanity as this? Rather let us have the timidity which dares not stand alone than the vanity that struts about with peacock airs beseeching a too busy world to admire the resplendence of its tail.

The medical writer should do as other people do who write books. The literary man studies models of style; he compares one model with another; he strives to acquire a taste and style of his own; he goes to school, in fact, as every person should who desires to become a scholar. Now if any subject requires for its proper handling a master of style, it is that of medicine. The field covered by it is so vast, the knowledge included within its range is so various, the problems calling constantly for solution are so profound, and the opinions expressed on every side are of such Babel-like confusion, that only a man of the clearest thoughts and the most logical precision of style should venture to write on a medical subject at all. The pitiable and disgraceful truth is that medical books are too often written, not for the purpose of stimulating thought and enlightening the mind, but with the mean object of advertising the name and personality of the writer to the world. There seems to be no way of preventing this miserable prostitution of literary methods to private ends. But we may, at least, call upon all those medical and scientific writers who aim at the instruction and enlightenment of their fellows to avoid the vulgar arts of those whose object is display, and to write with such simplicity and clearness that all who desire to learn may understand what it is they are being taught. Self-respect, as well as regard for the honour of a great profession, has a right to demand this measure of obedience to the accepted canons of taste.

